# Legionnaire’s disease, weather and climate

**DOI:** 10.2471/BLT.14.142299

**Published:** 2015-03-27

**Authors:** Ryota Sakamoto

**Affiliations:** a Hakubi Center for Advanced Researches, Kyoto University, 46 Shimoadachi-cho, Yoshida, Sakyo-ku, Kyoto 606-8501, Japan.

In the summer of 1976, at a convention for the American Legion, a mysterious outbreak of pneumonia affected 182 people, of whom 29 died. The spread of the infection appeared to be airborne, but it was not until the following year that the cause was identified as a bacterium. Legionnaire’s disease, as it is now known, is caused by inhalation of aerosols (fine particles or droplets), containing bacteria of the genus *Legionella*. This pattern of transmission means that the disease is likely to be affected by weather and climate, but the Intergovernmental Panel on Climate Change, (IPCC) did not include it in its recent report.^1^ This paper argues that Legionnaire’s disease should now be added to the IPCC’s list of important climate-sensitive health issues.

*Legionella* accounts for 2–15% of hospital admissions for community-acquired pneumonia, with a summer or autumn peak in incidence. Although *Legionella* seems be detected throughout the world, in many countries relevant laboratory tests are unavailable and the incidence of legionellosis is not known. According to population-based surveillance conducted in Ohio, United States of America, in 1997, the annual number of cases of legionellosis requiring hospitalization was estimated at 7 per 100 000 population.^2^ This incidence cannot, however, be generalized to other areas because transmission will be affected by local conditions.

*Legionella* is ubiquitous in the natural environment, especially in damp soil and water.^3^^–^^8^ Given that the organism is present more or less everywhere, what factors are responsible for occurrence of the disease – and are the same pathways responsible for both outbreaks and sporadic cases? *Legionella* is an intracellular parasite that multiplies inside host cells. In the natural environment, these cells include aquatic protozoa, and in humans, macrophages. When exposed to unsuitable conditions, (i.e. too cold or too dry) *Legionella* alters its metabolism and remains viable but not culturable. Water temperatures of 25–42 °C are ideal conditions for rapid growth.^7^ This explains why outbreaks of Legionnaire’s disease have often been linked to contaminated artificial water systems – especially air conditioning units in large buildings which use water for cooling.

Studies of associations between weather variables and sporadic cases of legionellosis suggest alternative potential exposure pathways. Associations have been reported between legionellosis and several weather variables,^9^^–^^15^ but the most consistent results relate to rainfall. Fisman et al. found that legionellosis was associated with rainfall 6–10 days before disease onset.^9^ This timing corresponds to the latent period between exposure to the pathogen and the development of symptoms. Several subsequent studies have identified small but statistically significant increases in the risk of legionellosis with increased rainfall after a lag time of one to two weeks.^10^^,^^11^^,^^13^^,^^14^

It is plausible that rainfall might affect exposure to *Legionella,* via a range of potential mechanisms. Contamination of reticulated drinking water is a possibility,^7^ but a one or two week lag time seems too short for this pathway. Another suggestion is that vehicles might produce aerosols containing *Legionella,* as they drive on wet road surfaces.^3^^,^^13^ Molecular matching of clinical and environmental samples is a promising approach that provides some support for this hypothesis.^4^

The environmental sources and global impact of legionellosis should now be reassessed. Being aware that *Legionella* is ubiquitous is not sufficient. It exists in the environment surrounding us, but which sources are the most important for human health? According to the IPCC, increases in heavy rainfall are projected as a result of global climate change.^16^ Climate change might increase the incidence of legionellosis through increased reliance on air conditioning systems, as well as through more subtle effects on bacterial ecology or airborne exposure pathways.
